# Staged Hybrid Repair of a Complex Type B Aortic Dissection

**DOI:** 10.3390/jcdd9090297

**Published:** 2022-09-06

**Authors:** Cristina-Maria Șulea, Csaba Csobay-Novák, Zoltán Oláh, Péter Banga, Zoltán Szeberin, Ádám Soltész, Zsófia Jokkel, Kálmán Benke, Máté Csonka, Eperke Dóra Merkel, Béla Merkely, Zoltán Szabolcs, Miklós Pólos

**Affiliations:** 1 Department of Cardiac Surgery, Heart and Vascular Center, Semmelweis University, Városmajor Str. 68, 1122 Budapest, Hungary; 2 Department of Interventional Radiology, Heart and Vascular Center, Semmelweis University, 1122 Budapest, Hungary; 3 Semmelweis Aortic Center, Semmelweis University, 1122 Budapest, Hungary; 4 Department of Vascular Surgery, Heart and Vascular Center, Semmelweis University, 1122 Budapest, Hungary; 5 Department of Anesthesiology and Intensive Therapy, Semmelweis University, 1122 Budapest, Hungary; 6 Department of Cardiology, Heart and Vascular Center, Semmelweis University, 1122 Budapest, Hungary

**Keywords:** type B aortic dissection, frozen elephant trunk, TEVAR, debranching, aortic team

## Abstract

Due to its heterogeneous clinical picture and lengthy evolution, the management of type B aortic dissection represents a clinical challenge, often calling for complex strategies combining medical, endovascular, and open surgical strategies. We present the case of a 45-year-old female who had previously suffered a complicated type B aortic dissection requiring a femoro-femoral crossover bypass and further conservative treatment. Seven years later, due to an aneurysmal development, a staged descending aortic management was strategized, beginning with the implantation of a frozen elephant trunk device due to an insufficient proximal landing zone for endovascular repair. However, the development of a distal stent graft-induced new entry complicated the dissection and led to the formation of a second false lumen, thus prompting an expedited hybrid reconstruction. We describe a hybrid repair strategy tailored to the patient’s particular aortic anatomic conformation, combining ilio-visceral debranching and thoracic endovascular aortic repair. Due to a lack of consensus on the ideal management strategy for type B aortic dissection, an individualized approach conducted by an experienced aortic team may generate the best outcome. The appropriate timing and planning of the intervention are the keys to successful results in complex type B aortic dissection cases with an elaborate anatomic conformation.

## 1. Introduction

Type B aortic dissection (TBAD) is a potentially life-threatening condition with a 13% in-hospital mortality rate in the acute phase [1]. Acute TBAD may be classified as uncomplicated or complicated, the latter being defined by the presence of aortic rupture, rapid aortic enlargement, visceral or limb malperfusion, refractory pain, or hypertension. The initial presentation of the dissection and its anatomic features are the main factors that direct the management strategy [2]. 

Endovascular management in the form of thoracic endovascular aortic repair (TEVAR) has become the preferred choice of treatment for acute TBAD. Although it may be used with satisfactory results in both uncomplicated and complicated cases, its deployment is highly dependent on aortic anatomy and the location of the primary entry tear. Therefore, a rigorous patient-tailored approach is essential, as a definitive treatment protocol has not yet been established. In selected complex cases of TBAD where the endovascular approach is not feasible, open surgical repair by the frozen elephant trunk (FET) technique has proven to be a safe therapeutic alternative, providing satisfying results in matters of technical success without increasing the operative risk [3,4]. The safe antegrade deployment of the self-expandable stent graft into the downstream aorta not only enables the coverage of entry points and the enlargement of the true lumen (TL) but also promotes the thrombosis of the false lumen (FL) [5]. As the FET technique may offer a practical landing zone for potential subsequent aortic endovascular interventions, this justifies its complementary use with TEVAR in the context of extensive thoracoabdominal aortic diseases.

We describe a case of total arch replacement by the FET technique for chronic TBAD with a complex anatomic conformation subsequently complicated by a new intimal injury which further led to a triple-barreled aortic dissection and prompted a hybrid repair resulting in a sub-total scaffolding of the aorta.

## 2. Case Presentation

In 2013, a 37-year-old female was admitted to the emergency unit citing two consecutive episodes of a sudden onset of severe, tearing back pain, the second one manifesting with irradiation along the spine into the right lower limb and being followed shortly by numbness and a loss of function of the extremity. She had a history of heavy smoking and severe arterial hypertension, which had been left uncontrolled for the past year. Upon physical examination, the right inferior limb presented signs of hypoperfusion, with no palpable femoral pulse. The patient exhibited marfanoid habitus (arachnodactyly, long limbs, tall and thin appearance, and scoliosis), but did not meet the clinical criteria for the diagnosis of Marfan syndrome according to the revised Ghent nosology (systemic score of 3) [6]. 

**Figure 1:**
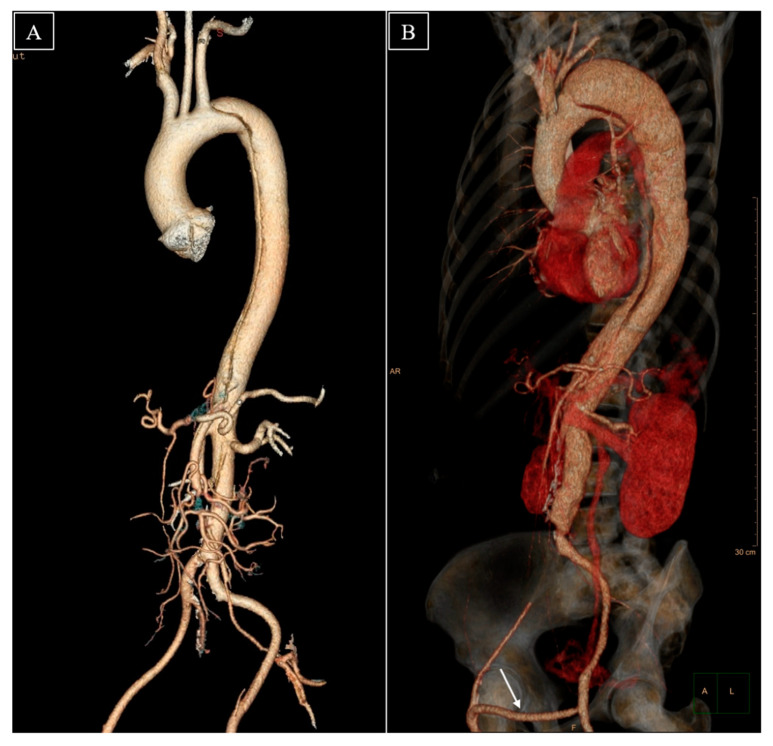
3D computed tomography angiography. (**A**) The TBAD in 2013. (**B**) The TBAD in 2020. The white arrow indicates the femoro-femoral crossover bypass graft implanted in 2013.

An emergency computed tomography angiography (CTA) uncovered a TBAD (Figure 1A) extending from just distal to the origin of the left subclavian artery to the aortic bifurcation and spreading bilaterally to the iliac arteries such that the lumen of the left common iliac artery was in continuation of the aortic FL, while a static occlusion by the dissection flap over the right common iliac artery ostium was noted. The celiac trunk, superior mesenteric artery, and left renal artery originated from the FL, whereas the right renal artery emerged from the TL, which was completely occluded below this level. The two aortic lumens communicated through multiple fenestrations in the intimal flap that resulted from the intimal stripping around the emergence of the aortic branches. 

An acute medical management protocol was instituted, consisting of continuous monitoring on the intensive care unit and antihypertensive measures. The imagistic findings prompted an emergency surgical revascularization procedure in the form of a left-to-right femoro-femoral crossover bypass procedure. As the dissection was not complicated by visceral malperfusion and the peripheral blood flow was satisfactory following the bypass procedure, the patient was subsequently put under a conservative treatment of arterial hypertension and was to be kept under systematic imaging surveillance. Control CTA investigations performed 2 and 3 months after the surgical intervention showed a competent bypass graft and no progression of the TBAD.

**Figure 2:**
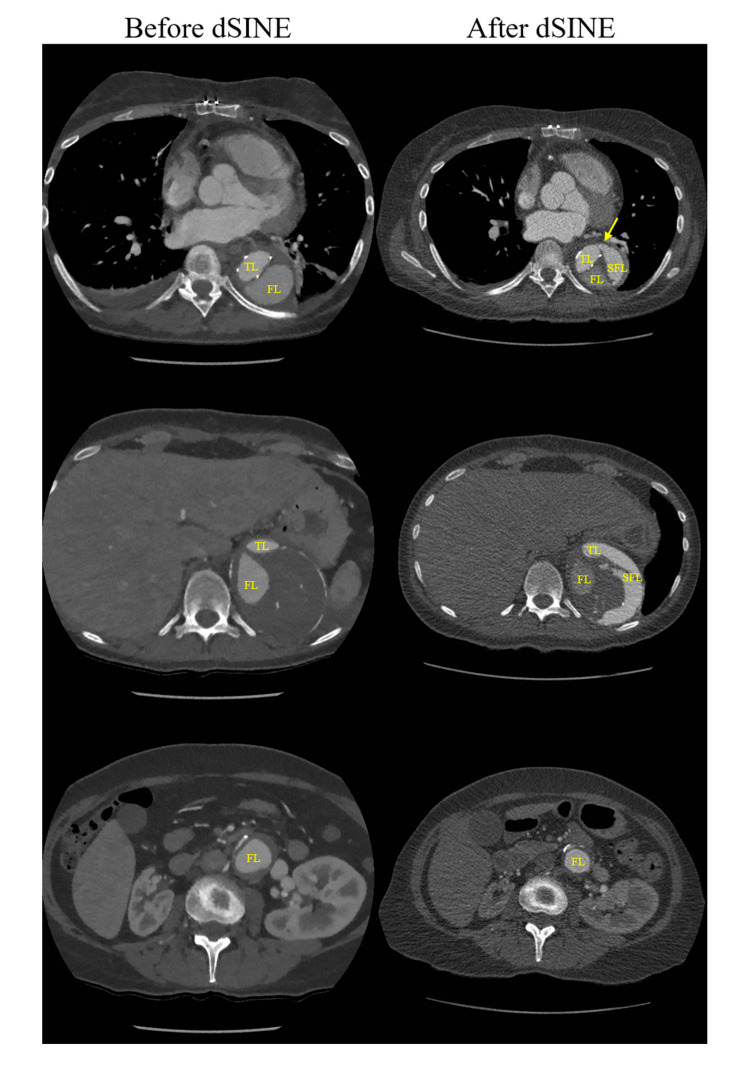
Postsurgical computed tomography angiography images comparing the conformation of the TBAD at different levels before and after the occurrence of the dSINE (marked with a yellow arrow), which determined the formation of a second false lumen (SFL). The true lumen (TL) is completely occluded distal to the emergence of the renal arteries. The initial false lumen (FL) exhibits partial thrombosis.

Following a years-long gap in the follow-up, the CTA performed in 2020 (Figure 1B) revealed the aneurysmal evolution of the TBAD, the proximal descending aorta reaching a maximum diameter of 72 mm in the axial plane (2 cm growth compared to 2013). The TL had progressively narrowed, while the FL exhibited gradual thrombosis. The crossover bypass graft was patent and the blood flow to the inferior extremities was sufficient. The localization of the primary intimal tear, nearly tangential to the emergence of the left subclavian artery, and the very close proximity of the great supra-aortic vessels to each other led to an inconvenient proximal stent graft landing zone, which was not favorable for total endovascular aortic repair. Therefore, a 26/28 mm Thoraflex™ Hybrid device (Terumo Aortic, FL, USA) was implanted in an effort to occlude the primary entry point and prevent further aortic dilation, while providing a base for the further endovascular repair of the descending aorta. The stent graft of the device was deployed inside the TL of the aorta in such a manner that it sealed the primary entry tear. The supra-aortic vessels were reimplanted individually into the designated branches of the device. The total operative time was 300 min, with extracorporeal circulation, aortic cross-clamp, and deep hypothermic circulatory arrest times of 205, 87, and 60 min, respectively. Postoperatively, no endoleak was noted. However, the patient’s initially favorable recovery was marked by an episode of newly onset severe thoracic pain that debuted approximately two weeks after the surgical intervention. A CTA showed a distal stent graft-induced new entry (dSINE) at the extremity of the Thoraflex™ stent graft, which determined a secondary dissection in the wall of the FL and resulted in a slight increase in aortic diameter (Figure 2).

**Figure 3:**
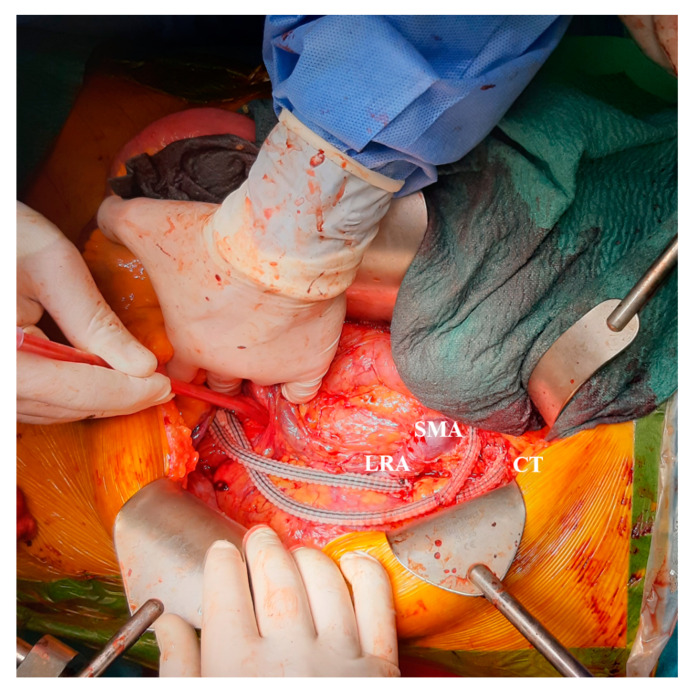
Intraoperative image showing the visceral debranching graft with its three branches supplying the left renal artery (LRA), the superior mesenteric artery (SMA), and the celiac trunk (CT).

**Figure 4:**
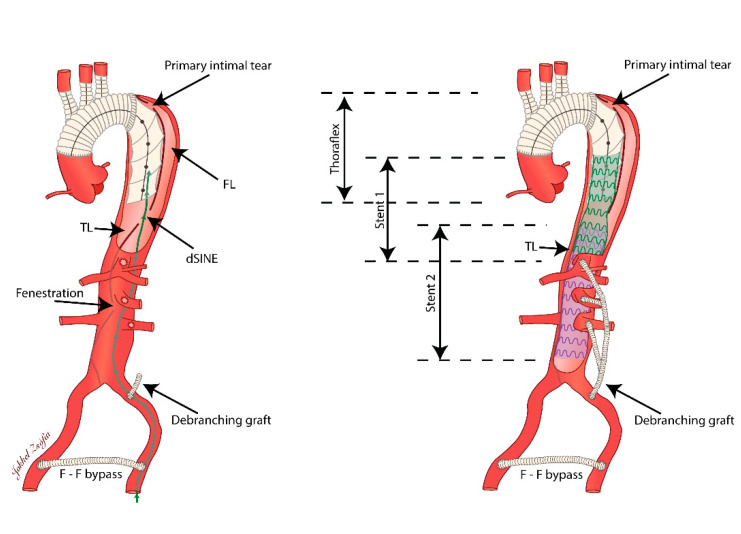
Illustrations depicting the endovascular intervention. The first stent graft (green) was guided into position along the route indicated by the green line (left femoral artery–aortic FL–dSINE–aortic TL). A second stent graft (purple) was placed in continuation of the first to restore the luminal conformation of the aorta. A visceral debranching graft was implanted beforehand to ensure proper blood supply to the left renal artery, superior mesenteric artery, and the celiac trunk. dSINE—distal stent graft-induced new entry; F-F—femoro-femoral; FL—false lumen; TL—true lumen.

**Figure 5:**
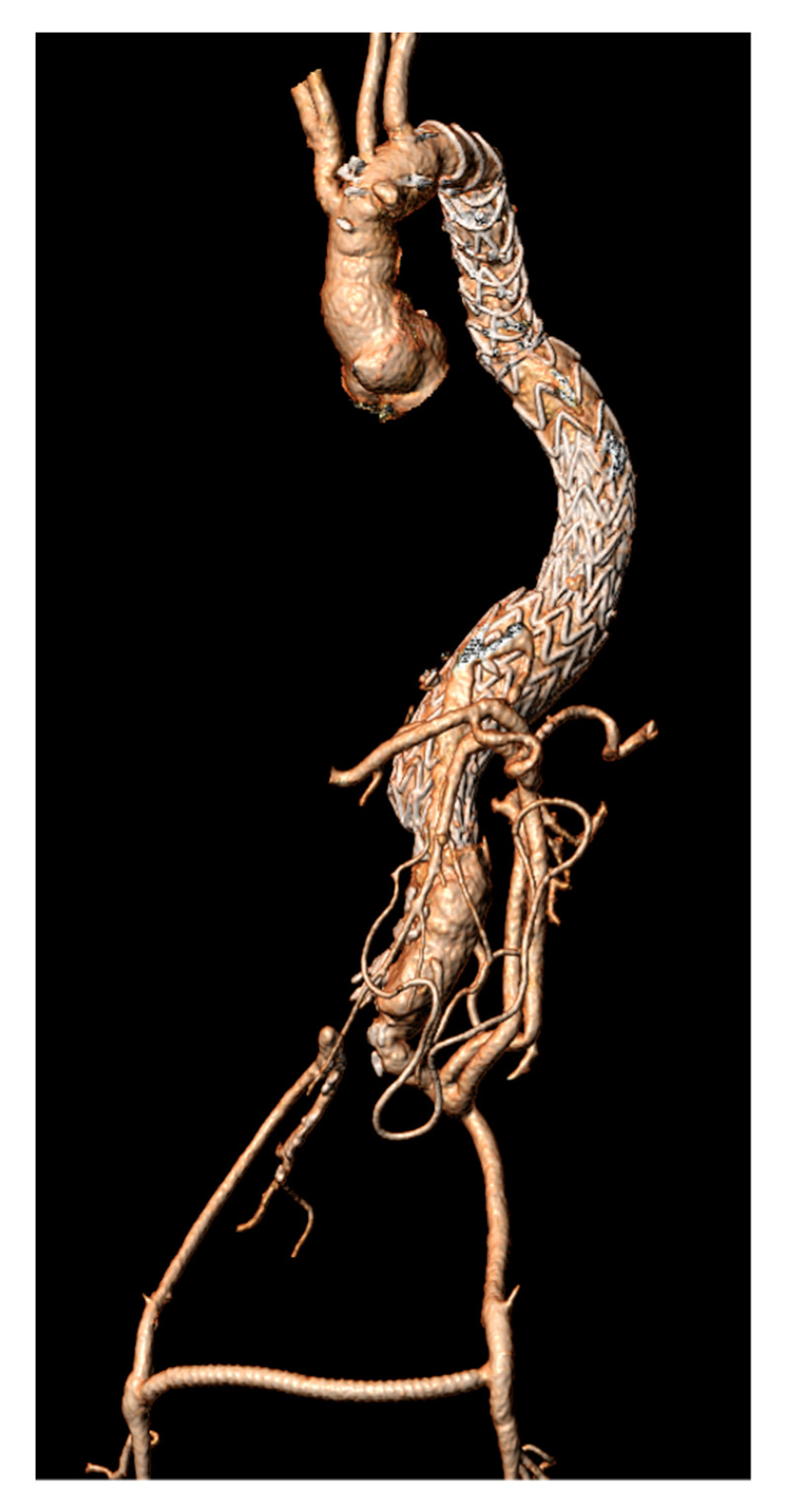
Postoperative 3D computed tomography angiography illustrating the final result after the hybrid reconstruction.

An emergency hybrid intervention was performed, consisting of a visceral debranching followed by thoracic endovascular aortic repair. Proper blood supply to the celiac trunk, superior mesenteric artery, and left renal artery was ensured through a custom-designed branched bypass graft attached to the left common iliac artery via left retro-peritoneal exposure (Figure 3). As the approach did not enable its revascularization by open means, the intraoperative decision was made to sacrifice the right renal artery. Next, after gaining vascular access via the left femoral artery, a guidewire was directed through the initial FL up to the abdominal segment of the aorta, where it was further guided into the TL through the dSINE (Figure 4). An aortic stent graft (Medtronic Valiant 30 × 200 mm, VAMF 30 30 C 200) was delivered along the guidewire and deployed into the TL in a piled-up manner to the stent of the Thoraflex™ device, thus reinforcing the entry tear closure and assuring the TL’s consolidation. A second stent graft (Gore C-TAG 40 × 200, TGMR40 40 20) was placed in continuation of the previous one, such that it landed in the infrarenal FL, restoring a luminal aortic conformation up to the proximity of the aortic bifurcation (Figure 5).

The patient was uneventfully discharged on postoperative day 9. Early postoperative 1-month and 2-month CTA investigations showed no increase in aortic diameter, fully patent stent and bypass grafts, and satisfactory aortic flow. A minor type 2 endoleak was noted originating from one of the segmental branches of the thoracic descending aorta. Both the excluded segments of the initial FL and the second FL exhibited progressive thrombosis. The patient is under careful observation with regular CTA examinations as the further dilation of the infrarenal FL may prompt the distal extension of the devices down to the level of the common iliac arteries.

## 3. Discussion

Complicated TBAD is associated with a significantly higher (16.1%) early mortality rate compared with uncomplicated TBAD (2.6%) [7]. Moreover, the presence of complications has been identified as an indicator of a higher in-hospital mortality risk and a poor late outcome [8]. Malperfusion syndromes represent the most frequent complication of TBAD, implicating the lower extremities in 25–30% of cases with aortic arch and/or thoracoabdominal aorta involvement [9]. Therefore, the timely recognition and management of TBAD cases associated with end-organ ischemia are paramount.

Due to a lack of consensus on the ideal management strategy for TBAD, a patient-tailored approach is the key to an optimal outcome. Endovascular management in the form of TEVAR has been rapidly gaining ground as a preferred therapeutic approach, producing improved results compared to open surgery [10,11]. Despite this, the procedure may not always be feasible. We resorted to an initial surgical treatment of the complications and a further conservative management in the setting of the acute TBAD as the localization of the entry tear and the morphology of the aortic arch were unsuitable for TEVAR.

Following an optimal medical treatment, at least one-third of patients will require surgery for aortic-related complications within 5 years of the initial TBAD [12]. Proximal aneurysmal degeneration of chronic TBAD increases the need for delayed intervention, thus escalating the risk of aortic adverse events, as corroborated by our report [2,13]. In such cases, a subsequent open surgical approach may be a suitable choice [14,15], with the FET technique having been previously described also as effective one-stage management for chronic TBAD [16]. In the context of our case, a staged hybrid approach to the extensive aneurysmal evolution of the descending aorta was considered to be the fittest alternative. However, the occurrence of the dSINE prompted the expedited execution of the second aortic intervention, underlining the importance of the team’s flexibility and availability for potential unpredicted procedures. TEVAR as a second-stage intervention following FET is an advantageous option, yielding favorable outcomes both as an elective redo procedure for postoperative complications and a staged approach to extensive aortic disease [17,18]. 

Although controversial, endovascular aortic repair techniques using branched or fenestrated stent grafts (BEVAR and FEVAR) have also yielded favorable results in chronic aortic dissections with aneurysmal development [19]. The use of an ilio-visceral bypass is considered a less optimal alternative, especially in young patients due to its modest long-term results [20]. However, our decision to use TEVAR in combination with abdominal debranching is justified firstly by the fact that BEVAR and FEVAR prostheses require lengthy production times, which we could not afford in the context of the alarming risk of an acute adverse aortic event. Secondly, the anatomic conformation of the aorta’s visceral segment, the occlusion of the TL at infrarenal level, and the lack of secondary access due to the occlusion of the right common iliac artery, did not allow for the implantation of either BEVAR or FEVAR. The particularity of our case resides in the complexity of the endovascular technique, reconstructing the luminal anatomy of the descending aorta in a labyrinthine fashion across the dSINE in the dissection flap.

It is crucial to note the important risk of neurological impairment (e.g., ischemic stroke and spinal cord ischemia) following extensive aortic interventions. Both the FET and TEVAR are associated with significant rates of neurological complications [21,22]. Through the necessity of repeated interventions, the risk is further maintained; therefore, appropriate planning and timing are essential. Such cases require significant surgical and interventional expertise and should be assigned to experienced aortic centers.

## 4. Conclusions

The management of complex type B aortic dissection remains a clinical challenge and often implies a staged hybrid approach. A successful outcome relies heavily on the expertise of the aortic team, who are capable of readjusting the management strategy and performing complex aortic interventions in acute settings. The further advancement of endovascular and hybrid techniques may unearth new possibilities in the management sphere of extensive type B aortic dissections.

## Data Availability

Not applicable.
